# The current level of shared decision-making in anesthesiology: an exploratory study

**DOI:** 10.1186/s12871-017-0386-3

**Published:** 2017-07-12

**Authors:** F. E. Stubenrouch, E. M. K. Mus, J. W. Lut, E. M. Hesselink, D. T. Ubbink

**Affiliations:** 1 Department of Surgery, Academic Medical Center, Amsterdam, The Netherlands; 2 Department of Anesthesiology, Academic Medical Center, Amsterdam, The Netherlands

**Keywords:** Shared decision-making, Patient education, Preoperative period, Surgical procedures, operative

## Abstract

**Background:**

Shared decision-making (SDM) seeks to involve both patients and clinicians in decision-making about possible health management strategies, using patients’ preferences and best available evidence. SDM seems readily applicable in anesthesiology.

We aimed to determine the current level of SDM among preoperative patients and anesthesiology clinicians.

**Methods:**

We invited 115 consecutive preoperative patients, visiting the pre-assessment outpatient clinic of the department of Anesthesiology at the Academic Medical Center of Amsterdam. Inclusion criteria were patients who needed surgery in the arms, lower abdomen or legs, and in whom three anesthesia techniques were feasible. The SDM-level of the consultation was scored objectively by independent observers who judged audio-recordings of the consultation using the OPTION^5^-scale, ranging from 0% (no SDM) to 100% (optimum SDM), as well as subjectively by patients (using the SDM-Q-9 and CollaboRATE questionnaires) and clinicians (SDM-Q-Doc questionnaire). Objective and subjective SDM-levels were assessed on five-point and six-point Likert scales, respectively. Both scores were expressed as percentages.

**Results:**

Data of 80 patients could be analysed. Objective SDM-scores were low (30.5%). Subjective scores of the SDM-Q-9 and CollaboRATE were high among patients (91.7% and 96.3%, respectively) and among clinicians (SDM-Q-Doc; 84.3%). Apparently, they appreciated satisfaction rather than SDM, being poorly aware of what SDM entails.

**Conclusion:**

The level of SDM in an outpatient anesthesiology clinic where preoperative patients receive information about various possible anesthesia options, was found to be low. Thus, there is room for improving the level of SDM. Some suggestions are given how this can be achieved.

## Background

Shared decision-making (SDM) is the process in which healthcare providers and patients decide together about the preferred treatment choice when more than one treatment option is available, using the best available evidence [1, 2]. SDM is one of the three pillars in the definition of evidence-based medicine [3]. The principle of evidence-based medicine has been widely accepted and includes appreciation of the situation and preference of the patient. However, many healthcare providers mainly focus on the finding and application of evidence, while the SDM-aspect tends to be neglected [3]. This became particularly clear from studies in surgical settings [4, 5].

There are several arguments that favour the application of SDM in various specialties. First, SDM is considered a moral and ethical principle [6]. Second, patients’ preferences may differ from the doctors’, and when there is equipoise between two or more different treatment options, patients’ preferences should be guiding the final choice [2, 7]. Third, SDM may reduce overdiagnosis and may lower surgical overtreatment [7, 8]. Fourth, research has shown that patients usually desire a more active role in decision-making [9, 10].

Currently there is little evidence regarding the extent to which SDM is applied in anesthesiology, although the vast majority of patients requiring anesthesia wishes to be involved in the decision-making process [11]. Furthermore, this specialty seems particularly suitable for SDM as it offers a range of equally effective anesthesia techniques for various patients undergoing surgery.

The aim of the present study is to determine the current level of SDM at the anesthesiology department of a university hospital during consultations between clinicians and preoperative patients.

## Methods

The hospital’s medical ethics review board approved the study and waived the need for full assessment, because the study did not have a serious impact on patient integrity or their treatment. This study was conducted in a tertiary care university hospital, in the pre-assessment outpatient clinic at the department of Anesthesiology. During this pre-assessment, patients scheduled for a surgical intervention are screened and informed about the options for per-operative anesthesia and post-operative analgesia.

We invited anesthesiologists, anesthesiologists in training, and anesthesia assistants who, by rotation, see these patients at the outpatient clinic to participate in this study. Clinicians did not receive any SDM-training prior to this study.

### Patient selection

A consecutive series of eligible patients was included. Patients should require surgery in the arms, lower abdomen or legs, for which three anesthesia techniques were feasible (i.e., general, spinal or epidural anesthesia or nerve block), and should have provided written informed consent. Patients under the age of 18, not able to participate in their decision-making process, or not able to comply and complete the questionnaires were excluded. Also patients already admitted and requiring re-interventions, as well as those presenting at the emergency department were not included.

### Sample size

For generalisability purposes we involved all staff members of the outpatient clinic (i.e., 21 care professionals, comprising anesthesiologists, anesthesiologists in training, and anesthesia assistants). For this descriptive part of the study, we planned to include an average of 5 patients per care professional to account for possible intra-clinician variation, as suggested in a previous study [5]. A sample size of 68 consultations would be sufficient to detect an intermediate effect size of 0.4 regarding the differences between the SDM-Q and Collaborate questionnaires, using a Mann-Whitney U-test with a 0.05 two-sided significance level and a 90% power.

### SDM-measures

All questionnaires used had been translated into Dutch previously [12]. The 5-item OPTION instrument was used to score the level of SDM objectively by independent observers (EMKM, JWL). These observers were experienced in judging clinician-patient encounters, but were not present during the consultations studied here. The five items are scored on a scale from 0; “the behavior is not observed” to 4; “the behavior is observed and executed to high standard” [13, 14] which means that total OPTION scores can range between zero and 20. We used the general description in the original manual to score each OPTION-item [15].

To assess the OPTION scores, the consultations between patient and clinician were audiotaped for analysis. The results of this instrument were used as primary outcome of the study.

The SDM-Q-9 is a validated, nine-item questionnaire. It was developed as a brief patient-reported instrument for measuring SDM in clinical encounters [16, 17]. The items are scored on a six-point Likert scale, ranging from “completely disagree” to “completely agree”. Hence, the total SDM-Q-9 scores vary between zero and 54. The 9-item SDM-Q-Doc questionnaire, in which the SDM-Q-9 questions are rephrased to reflect the clinicians’ point of view, aims to assess the clinician’s perspective of the SDM process in clinical encounters [17]. The SDM-Q-9 and SDM-Q-Doc questionnaires were used, because these are the most commonly applied tools to assess the patient-reported and doctor-reported levels of SDM.

We also applied the Collaborate scale, which was developed more recently as a ‘fast and frugal’, valid and reliable, patient-reported measure of the SDM process [18]. It has only three items addressing the effort made regarding patient involvement in the decision making process. The items are scored on a ten-point Likert scale, ranging from “no effort made” to “every effort made”. Thus, the total CollaboRATE scores can vary between zero and 30.

### Study conduct

Before the consultation patients were asked informed consent. During the consultation, which has a rather standard structure, the anesthesiology clinician asks about previous surgical interventions and the patient’s experience with the type of anesthesia during that procedure, and possible conditions that would interfere with possible anesthesia techniques. The clinician conducts a standard physical examination and proposes the possible anesthesia procedure, which is documented in the electronic medical record.

Directly after the consultation, the perceived level of SDM during the consultation was subjectively assessed by patients (using the SDM-Q-9 and CollaboRATE questionnaires) and clinicians (using the SDM-Q-Doc questionnaire). Finally, the investigators recorded the patients’ baseline characteristics using a short questionnaire: age, gender, education level, previous operations, possible preferences regarding the anesthesia technique based on previous experiences, and type of disorder.

### Statistical analysis

To ensure reliable assessment of the OPTION scores, three investigators initially scored ten random consultations to assess inter-observer agreement by calculating the kappa (κ) value. A κ-value expresses the level of agreement above chance. Kappa values above 0.8 denotes almost perfect agreement, between 0.8 and 0.6 substantial, between 0.6 and 0.4 moderate, and between 0.4 and 0.2 fair agreement [19]. If kappa was above 0.8 the remaining consultations were scored by only one investigator (EMKM).

The Statistical Package for the Social Sciences version 22 (IBM SPSS Inc., Armonk, NY, USA) was used to perform statistical analyses. The scores of all questionnaires were considered to have a non-normal distribution and were therefore presented as medians and interquartile ranges (IQR). Differences between SDM-Q-9 scores and CollaboRATE scores were analysed using a Mann-Whitney U test. Differences in SDM-Q scores between clinicians and patients were investigated using a Bland-Altman plot [20]. This plot shows the agreement between two different assays and offers the possibility to detect systematic differences between the assays and trends across the range of scores, if any. The scores of the OPTION^5^ instrument were also presented as box plots for each item as well as for the clinician groups separately, to detect possible differences in preferred levels of patient involvement. The SDM-Q-Doc scores were also presented as a box plot for each clinician.

To compare the results of individual clinicians, we only chose clinicians in whom at least five recordings were made to reliably detect any intra-clinician variation [5].

The responses to the SDM-Q-9, SDM-Q-Doc, CollaboRATE, and OPTION^5^ instruments were expressed as percentages of the maximum scores to allow comparison of the scores (0% = no SDM, 100% = optimum SDM). To analyse whether the different OPTION^5^- and SDM-Q-9 scores were due to variation among patients or among caregivers, we calculated intra-class correlation coefficients (ICC). A possible relationship between duration of the consultation and SDM-Q-9, SDM-Q-doc, and Collaborate scores, the OPTION^5^-score, clinicians’ background, and the patients’ age was analysed by means of multivariable linear regression analysis.

If the threshold of five patients per clinician was not reached, these data were not used to compare intra-clinician differences. However, these data were included in the overall analysis. If a questionnaire or audio-recording was missing, the data set was considered not complete. In these cases the subjective and objective data could not be compared.

## Results

**Fig. 1:**
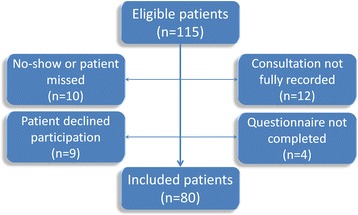
Flowchart of patient inclusion

Patient selection took place between September 2015 and February 2016. Based on the appointment list of the outpatient clinic, we eventually searched 115 possibly eligible patients as ten of these did not show up. Of the 105 remaining patients, 25 could not be included for several reasons (see Fig. 1), resulting in an inclusion rate of 76%. The 80 remaining consultations were performed by 21 clinicians; 3 anesthesiologists, 10 anesthesiologists in training, and 8 anesthesia assistants, aged 25–64 years and of whom 8 were men. The consultations had a mean duration of 12 min and ranged from 1.3 to 24.3 mins. This duration was not significantly related to the SDM-Q-9, SDM-Q-doc, Collaborate scores, or the clinicians’ background, but a significant positive association was found between the conversation duration and the OPTION score (*p* = 0.001), as well as a small but significant association with the patient’s age (*p* = 0.03). In 9 out of the 21 clinicians involved we were able to record at least five consultations.

Characteristics and preferences of included patients are shown in Table 1.

**Table 1:** Characteristics of included patients

	Included patients (*N* = 80)
Male	39 (48.8%)
Age (mean)	49.3 (SD14.9)
Specialty	
Surgery	11 (13.8%)
Urology	5 (6.3%)
Orthopaedics	52 (65.0%)
Plastic surgery	12 (15.0%)
Highest level of education	
Primary education	18 (22.5%)
Mean general education	18 (22.5%)
Secondary education	14 (17.6%)
Higher professional education	19 (23.8%)
University	11 (13.8%)
Underwent previous surgery	68 (85.0%)
Preference for anesthesia after previous surgery	
No preference	19 (23.8%)
Preference for the same type of anesthesia	37 (46.3%)
Preference for another type of anesthesia	12 (15.0%)
Anesthesia technique chosen	
General anesthesia	42 (52.5%)
Spinal anesthesia	18 (22.5%)
Peripheral nerve blockade	19 (23.8%)
No decision made	1 (1.3%)

### OPTION-scores

The original OPTION^5^ manual was found to be not specific enough to unequivocally assess the OPTION scores in this setting. Therefore, the evaluators discussed and specified how to score a certain level for each item. This adapted, more specific manual as developed is this study is presented in Table 2. After refining the manual, the kappa values rose above 0.80.

**Table 2:** Refined scoring definitions for the OPTION^5^ manual

Item	Description	Specification
1	The provider draws attention to, or re-affirms, a problem where alternate treatment or management options exist, and which requires the initiation of a decision making process. If the patient draws attention to the availability of options, and the provider responds by agreeing that the options need consideration, the item can also be scored positively.	0 – not observed
		1 – stating that several options exist
		2 – listing the options
		3 – equality of the options
		4 – is it clear / any questions
2	The provider reassures the patient, or re-affirms, that the provider will support the patient to become informed. The provider will support/explain the need to deliberate about the options.	0 – not observed
		1 – decide together
		2 – mention is it a difficult choice
		3 – will support irrespective of the choice of the patient
		4 – both options are o.k., depends on the preferences of the patient, provider has a supportive role
3	The provider gives information, or re-affirms/checks understanding, about options that are considered reasonable (including taking ‘no action’), to support the patient in understanding/comparing the pros and cons.	0 – no information
		1 – explaining pros and cons of one treatment
		2 – explaining pros and cons of more than one treatment
		3 – is it clear/any questions
		4 – ask the patient to repeat the information
4	The provider supports the patient to examine, voice, and explore his/her personal preference in response to the options that have been described.	0 – not observed
		1 – exploring one of the following items: preferences, concerns, expectations
		2 – exploring two of the following items: preferences, concerns, expectations
		3 – exploring all of the following items: preferences, concerns, expectations
		4 – integrates preferences/concerns/expectations for recommendation
5	The provider makes an effort to integrate the patient’s preferences as decisions are either made by the patient or arrives at by a process of collaboration and discussion.	0 – not observed
		1 – indicates need for decision
		2 – indicates need for decision based on the preferences of the patient
		3 – asking the patient if the patient is in agreement with the decision
		4 – provider indicates that the patient can abandon earlier choice
	Total score 0–20	
	Rescale 0–100	

**Fig. 2:**
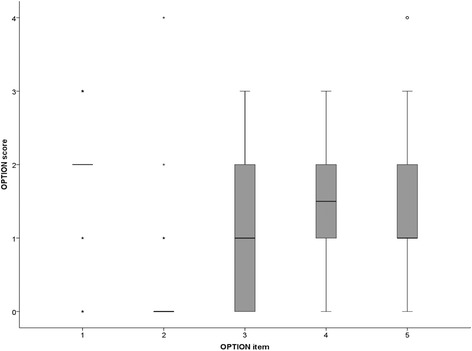
OPTION scores per item. OPTION items: 1 = Identifying a problem(s) needing a decision making process; 2 = the provider will support/explain the need to deliberate about the options; 3 = the provider list the options and explains the pros/cons; 4 = the provider explores the personal preference of the patient; 5 = the provider makes an effort to integrate the patient’s preferences as decisions are either made by the patient or arrives at by a process of collaboration and discussion. OPTION scores: 0 = not observed; 1 = there is a perfunctory or unclear attempt to perform the behavior; 2 = the behavior is performed at baseline skill level; 3 = the behavior is performed to a good standard; 4 = the behavior is performed to a high standard. Boxes represent values between 25th and 75th percentiles, whiskers the upper and lower adjacent values and the horizontal lines represent the median values. Outliers are displayed as asterisks

Overall, the objectively scored SDM levels using the OPTION instrument were low. Mean total OPTION-score was 30.5% (SD 10.5%). Figure 2 shows the OPTION-scores per item. Item 2 “justify the work of deliberation as a team” was rated as “not observed” in almost all consultations (77/80). The highest median score (2; baseline skill level) was found for item 1 “naming options”, but with a large variation. Item 3 showed the largest variation, representing differences in the amount of information given about the anesthesia options.

**Fig. 3:**
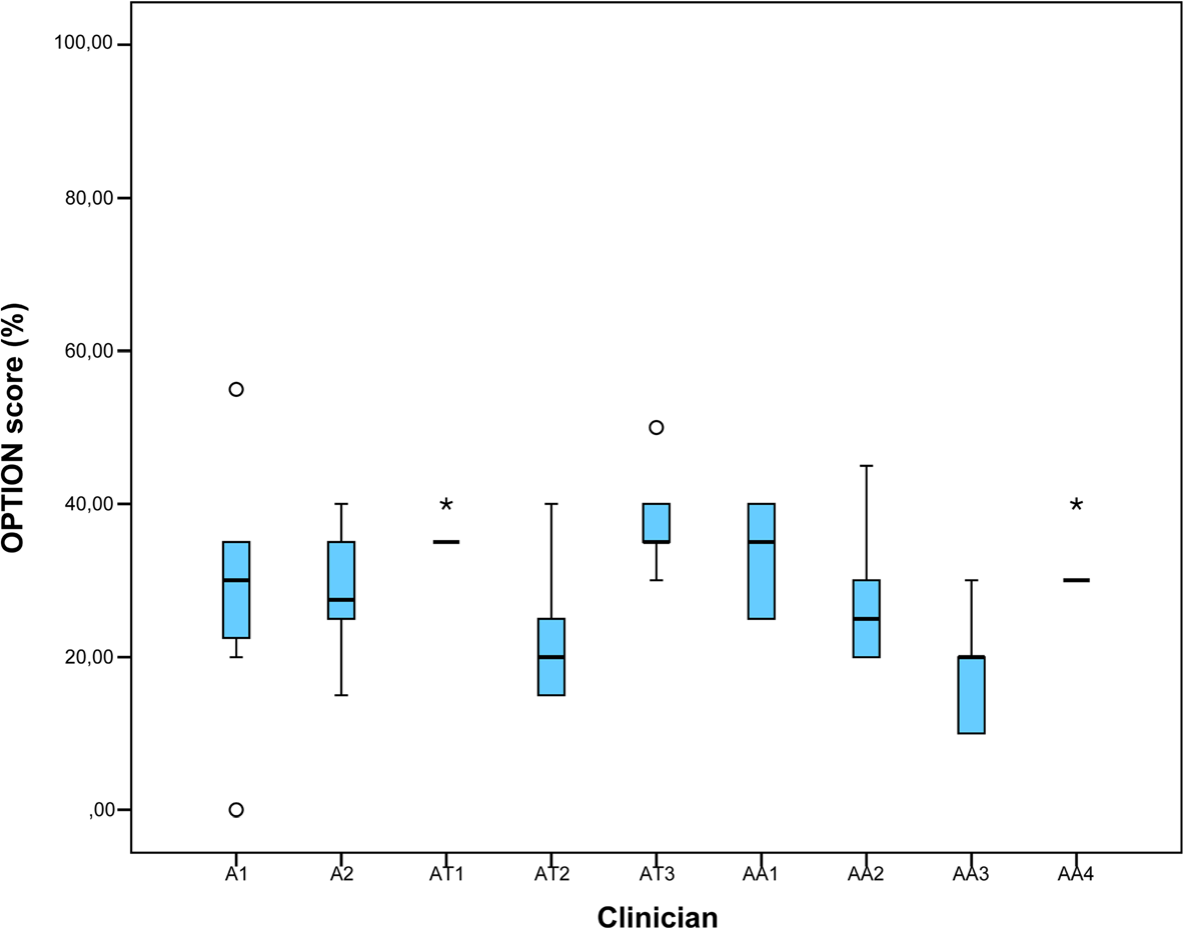
OPTION scores per clinician. ‘A’ stands for anesthetists, ‘AT’ stands for anesthetists in training and ‘AA’ stands for anesthesiology assistant

Figure 3 shows the mean OPTION-scores per clinician. OPTION-scores were low, ranging from a median score of 20% to 35%, and did not substantially differ among the groups of clinicians.

### SDM-Q-9, SDM-Q-doc and CollaboRATE scores

**Fig. 4:**
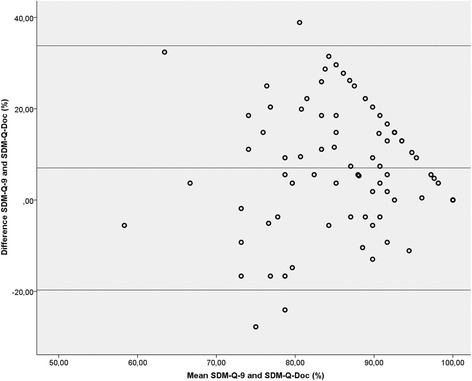
Bland-Altman plot of the differences between SDM-Q-9 and SDM-Q-Doc scores. The middle horizontal line indicates the mean difference between SDM-Q-9 and SDM-Q-Doc, while the upper and lower horizontal lines show the 95% limits of agreement

Subjectively perceived SDM-scores among patients and clinicians were high. Median SDM-Q-9 score was 91.7% (IQR 83.3–100) in patients and 84.3% (IQR 74.3–90.5) in clinicians. Patients scored significantly higher than clinicians (*P* < 0.001). Figure 4 shows the relation between the SDM-Q-9 and SDM-Q-Doc scores. Again, patients scored systematically higher than clinicians (on average 7.1%, 95% CI 19.6 to 33.8%). CollaboRATE scores (96.3%; IQR 88.9–100.0) were slightly but significantly (*P* = 0.031) higher than the SDM-Q-9 scores (91.7%; IQR 83.3–100.0).

### SDM-Q-9, SDM-Q-doc and CollaboRATE scores per clinician

**Fig. 5:**
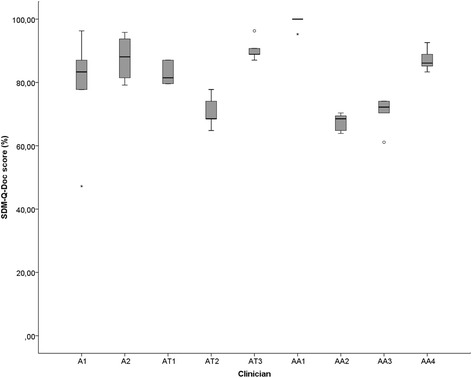
SDM-Q-Doc scores of each of the clinicians. ‘A’ stands for anesthetists, ‘AT’ stands for anesthetists in training and ‘AA’ stands for anesthesiology assistant

We found an ICC of 0.16 between caregivers and OPTION^5^-scores and an ICC of 0.06 between caregivers and SDM-Q-9 scores, indicating that the variance was mainly due to differences among patients, rather than among caregivers. The SDM-Q-Doc scores of the nine clinicians in whom five consultations were recorded are shown in Fig. 5. The clinicians’ scores were generally high; ranging from a median of 68.5% to 100.0%, and did not differ substantially among them. Patients (SDM-Q-9) also scored high, ranging from 72.2% to 100.0%, irrespective of the clinician involved. The same was true for the CollaboRATE scores, varying between 81.5% to 100.0%.

## Discussion

In an era of patient-related outcome measures (PROMs) and patient-related expectation measures (PREMs), shared decisions between doctors and patients are of crucial importance. Evidence suggests that doctors are but faintly aware of what matters most to their patients in the perioperative setting [2]. Shared decision enables doctors to gain more insight into the patients’ individual demands and expectations. For many patients scheduled for surgery, several anesthesia techniques are feasible to choose from. Hence, the patient could and should have a voice in this decision.

However, this study showed that in preoperative patients in whom a decision about the anesthesia technique is to be made the level of patient involvement during pre-assessment by anesthesiology clinicians is low. In contrast, patients and clinicians subjectively perceived the consultation as satisfactory. One could argue about the need for SDM when patients and clinicians are satisfied with the current situation. However, the amount of evidence is growing that SDM contributes to a better quality, safety and cost-effectiveness of care, [1, 7, 21] and has been acknowledged as a moral and ethical requirement of present-day care [6]. This awareness is still burgeoning among physicians and patients.

The low level of patient involvement in decision-making as found in this study is in agreement with earlier studies in various other clinical settings, [5, 13] so there is room for improvement. Clearly, the majority of anesthesiology clinicians were still insufficiently aware of what SDM entails. Although they generally inform patients about the options and (some of) their pros and cons, they hardly invite patients to share in the decision-making process, even though more than one anesthesia option is feasible. This requires a change of attitude from informing and advising the patient what should be done towards showing the patients they have an important role in the decision-making process and engaging them in this collaboration.

Obviously, anesthesiologists are experts in their medical field, but patients are the better expert as to their values, goals and preferences when more options are feasible and properly explained. Thus, SDM may be fostered by explicitly stating patients have a voice in the decision-making process, explaining the pros and cons of each anesthesia option, supporting them to express their values and preferences regarding the types of anesthesia, and deliberate these options together to reach a final - shared - decision [22].

One of the explanations for the discrepancy observed between the subjective and objective appreciations of the level of SDM is that patients and clinicians often express their degree of satisfaction with the consultation rather than the perceived level of SDM. For example, patients judge subjective aspects, like the tone of voice and amount of empathy of the clinician, the way they felt during the consultation, etc. [23, 24]. When patients are not familiar with the concept of SDM, they probably tend to involve the factors mentioned above to a greater extent in their assessment. Second, the subjectively appreciated level of SDM can be higher than what is observed objectively, since assessment of the levels of SDM by patients and clinicians may be biased because of leniency and gratitude [18]. Furthermore, clinicians usually underestimate the amount of information patients desire and spend less time on the discussion about therapy than patients would appreciate [9, 11, 25]. Therefore, clinicians should discuss the patients’ preferred level of SDM in their consultations. If the desired level of involvement remains unclear, it is preferable to use a high level of SDM, as this was found not to impair the satisfaction of the patient [11].

Patients were found to score higher SDM levels than clinicians. Earlier research showed that patients are more willing to score maximum scores compared to clinicians [26]. The CollaboRATE instrument resulted in higher scores than the SDM-Q-9 tool. Although this difference was statistically significant, both scores were still much higher than the objectively scored level of patient involvement using the OPTION instrument. This indicates that the CollaboRATE instrument, developed as a ‘fast and frugal’ tool to assess the level of SDM, [18] is also strongly biased by how much patients know what SDM really is. If they are ignorant about this, the CollaboRATE scores tend to be erroneously high, as do the SDM-Q-9 scores. Thus, the OPTION instrument seem to more accurately reflect the actual level of patient involvement. The substantial variation in item 3 scores may be explained by the fact that a large proportion of patients had been operated before. Therefore, they probably needed less information about the options. Besides, some recordings were short, because the clinician started the audio-recording after the patient’s physical exam, whereas others recorded the whole visit, in which the exam was alternated with the conversation. This may explain the variation in consultation duration. Apparently, a better information and involvement of the patient as to the decision-making resulted in a longer duration of the conversation as well as a higher OPTION-score.

The SDM-Q-Doc showed less intra-clinician variation than the SDM-Q-9. This is likely because physicians perform their consultations in a routine, unvarying way, and therefore with a similarly constant level of SDM. Different patients have different opinions and therefore the level of SDM may differ among patients, resulting in a greater intra-clinician variation.

Some limitations of this study should be mentioned. We did not reach the intended five patients per caregiver. However, this number is merely a rule of thumb used in other studies. As this was merely used for a descriptive part of the study (i.e. assessing the level of patient involvement in SDM), it was not used for a sample size calculation. Moreover, in other similar studies using the OPTION scale, sample sizes the number of rated consultations per study ranged from 8 to 352, averaging 95 [5, 13, 14]. Despite this relatively small patient sample, it is not likely that a larger sample size would have resulted in a different conclusion.

The care professionals varied widely as to their background. This is common practice in pre-assessment clinics. Although we could not actually test differences in SDM levels between these groups because of small subgroup sizes, it is unlikely that the differences in background would have led to differences in the level of SDM. None of the clinicians were trained and did not receive SDM training during this inclusion period. Hence, they could not have changed their consultation technique. Furthermore, we did not instruct them how to apply SDM. In addition, the patient sample used seems generalizable because it is not likely that excluded patients were different from those analysed, as they were excluded for mere technical reasons or time constraints. Initially, despite the existing manual, it was hard to achieve an acceptable inter-observer reliability due to differences in interpretation of the conversations. For this reason the manual was refined. This was also found to be necessary in a previous study [5]. However, there is no reason to assume that the refined manuals deviate from the interpretation as intended by the original authors.

Audiotaping the consultations made clinicians aware of being recorded. This may have stimulated their effort to apply SDM in their consultations and might have overestimated the level of SDM we observed. However, earlier studies showed that the recording of consultations does not influence the clinicians’ behavior because they quickly forget that the consultation is being recorded [27, 28].

The vast majority of included patients had undergone surgery before. Most of them stated to have a preference for a particular anesthesia technique. Because these patients were likely to be better aware of the possibilities, the level of SDM could have been higher. However, our results do not support this and might have been even worse if more patients had contributed who needed surgery for the first time.

## Conclusion

The level of SDM in an outpatient anesthesiology clinic where preoperative patients receive information about various possible anesthesia options, was found to be low. As long as the personal preferences of patients are influenced by the expert opinion given by the caregiver, SDM falls short of the intended purpose. Thus, there is room to improve the level of SDM. For example: decision aids to better inform patients about possible anesthesia techniques and to invoke their preferences, [29] option grids for care professionals to support the SDM process in the consultation room, [22] and SDM e-learning modules to instruct patients and care professionals how SDM should be performed in clinical practice [30]. At this moment a patient decision aid and option grid are currently being developed explaining patients about the anesthesia and analgesia options.

## Abbreviations

ICC: intra-class correlation coefficients
IQR: interquartile ranges
PREMs: patient-related expectation measures
PROMs: patient-related outcome measures
SDM: shared decision-making
Κ: kappa
